# *ADAMTS1* and *HSPG2* mRNA levels in cumulus cells are related to human oocyte quality and controlled ovarian hyperstimulation outcomes

**DOI:** 10.1007/s10815-019-01659-8

**Published:** 2020-01-23

**Authors:** Yerong Ma, Jiamin Jin, Xiaomei Tong, Weijie Yang, Peipei Ren, Yongdong Dai, Yibin Pan, YinLi Zhang, Songying Zhang

**Affiliations:** 1 Assisted Reproduction Unit, Department of Obstetrics and Gynecology, Sir Run Run Shaw Hospital, School of Medicine, Zhejiang University, No. 3 Qingchun East Road, Jianggan District, Hangzhou, 310016 China; 2 Key Laboratory of Reproductive Dysfunction Management of Zhejiang Province, No. 3 Qingchun East Road, Jianggan District, Hangzhou, 310016 China

**Keywords:** *ADAMTS1*, *HSPG2*, Cumulus cells, PCOS, COH

## Abstract

**Purpose:**

The study investigated potential correlations between the expression levels of *ADAMTS1* and *HSPG2* in cumulus cells (CCs) and controlled ovarian hyperstimulation (COH) outcomes.

**Methods:**

RT-PCR was used to determine *ADAMTS1* and *HSPG2* mRNA levels in mice CCs at different timepoints (0, 4, 8, 12, and 16 h) after human chorionic gonadotropin (hCG) injection, and in CCs after RNAi treatment. Women with polycystic ovary syndrome (PCOS) (*n* = 45) and normal ovulatory controls (*n* = 103) undergoing IVF/ICSI were recruited. Relative *ADAMTS1* and *HSPG2* mRNA levels were measured by RT-PCR. Moreover, correlations of *ADAMTS1* and *HSPG2* levels with COH outcomes were analyzed.

**Results:**

At different timepoints after hCG treatment, *ADAMTS1* mRNA had the highest level at 12 h, whereas *HSPG2* showed opposite profiles to *ADAMTS1* with the lowest level at 12 h. *HSPG2* expression was upregulated after *ADAMTS1* RNAi treatment The PCOS group had higher *HSPG2* and lower *ADAMTS1* expression levels than controls. In normal ovulatory women (control group), a higher expression of *ADAMTS1* and lower expression of *HSPG2* were associated with more mature oocytes, transplantable embryos, and good quality embryos, whereas higher transplantable embryo rates and good quality embryo rates were obtained only with lower *HSPG2* expression. ROC curves showed the co-measurement of *ADAMTS1* and *HSPG2* had a better predictive power than separate analyses.

**Conclusion:**

The dynamic profiles of *ADAMTS1* and *HSPG2* were inversely correlated in CCs. In PCOS and normal ovulatory patients, higher *ADAMTS1* and lower *HSPG2* expression levels in CCs were related to better COH outcomes.

**Electronic supplementary material:**

The online version of this article (10.1007/s10815-019-01659-8) contains supplementary material, which is available to authorized users.

## Introduction

Ovulation, stimulated by a luteinizing hormone (LH) surge, is a dynamic series of events that includes oocyte meiosis resumption, cumulus expansion, follicle rupture, and cumulus oocyte complex (COC) release. LH mainly triggers the ERK1/ERK2 signaling cascade in granulosa cells (GCs) to induce the expression of many genes crucial for ovulation, oocyte meiosis, and extracellular matrix (ECM) remodeling [1]. Many of these ERK1/ERK2-dependent genes, including *AREG*, *ADAMTS1*, and *CITED2,* in GCs were associated with oocyte quality and IVF outcomes [2–7].

ECM remodeling is characterized by the degradation of localized ECM components, including versican, laminin, collagen IV, perlecan, and fibulin [2, 8–12]. ECM remodeling occurs throughout the whole ovulation process. For example, cumulus cell (CCs) expansion includes hyaluronan-rich ECM after an LH surge. Furthermore, successful ovulation requires precise ECM remodeling to ensure follicle rupture [11, 13–16].

*ADAMTS1* (a disintegrin and metalloprotease with thrombospondin type 1 motif 1), a member of the proteinase family, has catalytic activity against proteoglycans (such as aggrecan and versican) to remodel the ECM [2, 8, 14, 17–22]. Using an ADAMTS1 knockout mouse model, it was convincingly shown that *ADAMTS1* plays key roles in female reproduction [3]. *ADAMTS1* null mice have morphologically abnormal ovaries, reduced numbers of ovulated oocytes, and a reduced fertilization rate. Compared with *ADAMTS1*^±^ mice, *ADAMTS1*^−/−^ mice have high levels of versican, which is undetectable in *ADAMTS1*^±^ ovaries, indicating that the processing of versican by *ADAMTS1* is involved in ovulating follicle remodeling [14]. This indicates *ADAMTS1*, and versican levels are closely associated with oocyte number and competence. In recent years, several investigators have demonstrated a close correlation between *ADAMTS1* levels in human CCs or follicle fluids and impaired oocyte quality in polycystic ovary syndrome (PCOS) patients [23–26]. Consistent with *ADAMTS1*^−/−^ mice, *ADAMTS1* levels in CCs were decreased in PCOS patients, which might contribute to abnormalities, such as low fertilization rate and cleavage rate [4, 7, 26]. To date, no study has evaluated the relationship between *ADAMTS1* level and controlled ovarian hyperstimulation (COH) outcomes in normal ovulatory patients.

Perlecan/heparan sulfate proteoglycan 2 (*HSPG2*) encodes perlecan, a substrate highly expressed in ovarian ECM that is potentially cleaved by *ADAMTS1* [27]. Perlecan is an extracellular proteoglycan involved in tumor angiogenesis, proliferation, and invasion. It is also a potential ECM substrate of *ADAMTS1* during ovulation [27–29] and is involved in the stabilization of other molecules, permeability of the glomerulus to macromolecules, and cell adhesion [8, 30–33]. It is a potent inhibitor of smooth muscle cell proliferation and is therefore thought to help maintain vascular homeostasis [32, 34–36]. It also promotes growth factor activity, thereby stimulating endothelial growth and regeneration [13, 32, 35, 37]. There have been limited studies regarding the function of *HSPG2* in reproduction. One study showed that the quantity of perlecan in follicular fluid with fertilized oocytes was significantly greater than that with non-fertilized oocytes from the same patient [38]. Furthermore, low levels of perlecan were observed in PCOS patient follicular fluid [38]. However, whether *HSPG2* mRNA levels in human CCs are associated with oocyte quality is unknown. Thus, we investigated whether the mRNA levels of *ADAMTS1* and *HSPG2* in human CCs indicated oocyte quality.

In this study, we investigated the timing of the expression of *ADAMTS1* and *HSPG2* during ovulation by RT-PCR using mouse CCs following hCG injection. We found the opposite expression pattern of *ADAMTS1* and *HSPG2* at the mRNA level during ovulation. To determine the effect of *ADAMTS1* on *HSPG2* expression, we knocked down *ADAMTS1* in mouse COCs. We found that *ADAMTS1* RNAi increased *HSPG2* expression in CCs. Thus, we investigated whether *ADAMTS1* and *HSPG2* mRNA levels were related to COH outcomes. Therefore, we performed RT-PCR to compare *ADAMTS1* and *HSPG2* mRNA levels between PCOS patients and normal ovulatory women and confirmed the inverse pattern and predictive power of the two molecules. Last, in normal ovulatory women, the correlations between *ADAMTS1* or *HSPG2* mRNA level and COH outcomes (oocyte number, oocyte quality, and embryo developmental potential) were intensively investigated.

## Materials and methods

### Mouse mural GCs and CCs collection

C57BL/6 mice were obtained from the Zhejiang Academy of Medical Science, China. Animal care and experimental procedures were conducted in accordance with the Animal Research Committee guidelines of Zhejiang University.

Female wild-type C57BL/6 mice at postnatal day (PD) 23 were primed with 5.0 IU pregnant mare serum gonadotrophin (PMSG). And 48 h later, mice were injected with human chorionic gonadotropin (hCG) for different treatment times (0, 4, 8, 12, and 16 h). At these timepoints, ovaries were dissected, and follicles were punctured using needles. Then, mural GCs were harvested directly and CCs were obtained by removing the oocytes mechanically from COCs.

### Mouse COCs collection, RNAi, and in vitro maturation (IVM)

Enclosed COCs were collected by puncturing the ovaries from mice with 5 IU PMSG treatment for 48 h. IVM medium (Easy Check, China, M2115) with 2.5 μM milrinone (MCE, USA, HY-14252) was used to inhibit CCs expansion during collection and culture with siRNA. Prior to COCs collection, specific siRNAs (5′-GGAAGTACTGTGAAGGCAA-3′) (RIBOBIO, China, S181130134515) for the mouse *ADAMTS1* gene and a negative control at a final concentration of 50 pmol/ml were mixed with Lipofectamine™ 3000 Transfection Reagent (Thermo Fisher Scientific, USA, L3000015) for 30 min and then added into the cultured IVM medium containing 2.5 μM milrinone. The COCs were transferred to the RNAi medium and cultured for 24 h in a 5% CO_2_ incubator at 37 °C. After transfection with siRNA or negative control for 24 h, COCs were transferred and allowed to resume meiosis in new fresh IVM medium (Easy Check, China, M2115) without milrinone and then cultured in a 5% CO_2_ incubator at 37 °C for 12 h. The CCs were harvested from 10 matured COCs by a mechanical method for RNA isolation.

### Patient selection

This study included 148 women undergoing IVF/ICSI because of PCOS (*n* = 45; ICSI, *n* = 5; IVF, *n* = 40) or tubal factor or male factor (*n* = 103, ICSI, *n* = 23; IVF, *n* = 80) at the Reproductive Center of Sir Run Run Shaw Hospital affiliated to Zhejiang University from July 2017 to June 2018. The experiment was certified by the ethics committee of Sir Run Run Shaw Hospital affiliated to Zhejiang University (20190215–2). All patients signed informed consent regarding the collection of CCs.

Of all patients undergoing IVF/ICSI, we selected patients who were between 20 and 35 years old. Our study did not affect clinical treatment options, but we only chose patients who were treated with a long protocol. Briefly, mid-luteal gonadotropin-releasing hormone (GnRH) agonist (Ferring) and ovarian stimulation with a daily subcutaneous dose of hMG (Menogon, Ferring) were started on the third day of the menstrual cycle. When the leading follicles reached 18 mm in diameter, women received hCG (Merck, Serono). Patients with a minimum of seven large follicles ≥ 14 mm at the final ultrasound before oocyte retrieval were invited to attend.

At the same time, patients who did not meet the follow-up criteria were excluded. Inclusion criteria for normal ovulatory women (*n* = 103) were age > 20 and < 35 years old, regular menstrual periods between 23 and 35 days, and IVF/ICSI indication for tubal or male factor. Patients were excluded for any of the following conditions: (1) abnormal endocrinology, such as polycystic ovary syndrome or premature ovary failure; (2) day 3 serum FSH level > 10 IU/l; (3) ovarian hyperstimulation syndrome (OHSS); or (4) suffering from other diseases (cardiovascular, pulmonary, liver, or kidney disease).

Based on the Rotterdam criteria [39], patients with PCOS and two of the following criteria were included: (1) rare ovulation or anovulation; (2) hyperandrogenism or clinical manifestations of hyperandrogenism (such as hairy, acne); and (3) polycystic ovarian changes. Patients with other causes of hyperandrogenism (such as hyperprolactinemia and thyroid diseases, congenital adrenal cortical hyperplasia, Cushing’s syndrome, androgen-secreting tumors, 21-hydroxylase deficiency atypical adrenal cortical hyperplasia, and exogenous androgen use) were excluded.

### Human CCs collection

Aspiration of the oocytes was performed transvaginally by ultrasound guidance 36 h after hCG administration. Immediately after the isolation of COCs, professional staff cut part of the cumulus mass off. CCs from all 18–20 mm follicles of a patient were pooled. Erythrocytes were removed by adding erythrocyte lysis buffer (EL-buffer, Qiagen, Germany). Then, CCs were washed with 1 ml phosphate-buffered saline (PBS) twice and centrifuged for 10 min at 800 x g to form a pellet. The pellet was collected and stored at − 80 °C until RNA extraction.

### Human mural GCs collection

Human mural GCs were isolated from the follicular fluid by a pipette, and erythrocyte lysis buffer was added to remove erythrocytes. Similar to CCs, mural GCs were washed with PBS twice and centrifuged for 10 min at 800 x g to form a pellet. The pellet was collected and stored at − 80 °C until RNA extraction. Mural GCs from all 18–20 mm follicles of a patient were pooled.

### RNA isolation, reverse transcription, and RT-PCR

Total RNA was isolated using the RNeasy Plus Micro Kit (Qiagen, Germany, 74,034) according to the manufacturer’s protocol. Then, RNA concentration was determined by NanoDrop 2000 (Thermo Fisher) and cDNA was obtained from total RNA (50 ng) of each sample by reverse transcription using oligo (dT)_15_ and reverse transcriptase (Promega, USA, A3500), according to the manufacturer’s instructions, at 42 °C for 15 min, followed by 95 °C for 5 min in 20 μl total volume.

Quantitative real-time PCR was performed using SYBR Green Master Mix (ABI, Germany, DBI-2044) in an Applied Biosystems 7500 Real-Time PCR System. An aliquot (10%) of cDNA was subjected to 40 amplification cycles of PCR with the primers listed in Supplementary Table 1. Cycling parameters were one cycle for 2 min at 50 °C, one cycle for 10 min at 95 °C, 40 cycles at 95 °C for 15 s, and 60 °C for 20 s. For each experiment, three replicates were included in each qPCR reaction. We performed melting curve analysis at the end of each run to ensure a single amplicon.

The relative levels of endogenous β-actin mRNA (ACTB) mRNA were used as an internal control [40], and data were analyzed using the 2^-(△△Ct)^ method. Only samples whose cycle threshold (Ct) values of ACTB were between 21 and 22 were included in the analysis.

### Assessment of oocyte and embryo quality

Regarding clinical outcomes, the development conditions of oocytes and embryos were observed and recorded by professional staff. MII oocytes with their corresponding polar bodies and fertilized oocytes with pronuclei were observed at 4–6 h and 16–18 h after insemination, respectively. Good quality embryos and transferable embryos were judged according to a cleavage embryo scoring system [41] at day 3. Professional staff observed the number of cleavage spheres, their symmetry, cytoplasmic morphology, and the number of fragments produced during division. According to these parameters, the embryos were divided into four grades. Grade I–II embryos have equally sized blastomeres with 0%–20% fragmentation. Grade III embryos have unequal sizes and 20% fragmentation. Grade IV embryos have low developmental potential with an abnormal appearance. The grade I–II embryos were recorded as good quality embryos, and grade I–III embryos were recorded as transferable embryos. Only transferable embryos were frozen on day 3, after the assessment of embryo quality. Concurrently, grade IV embryos were discarded. When the patient had reached the condition of embryo transfer, two embryos were thawed and transferred, including endometrial thickness between 8 and 12 mm, E2 > 100 IU/L, and LH <10 IU/L. High-level embryos were transferred as a priority. For implantation, we included only the first transfer from each patient, and all embryos transferred were vitrified-warmed.

The fertilization rate of IVF = the number of zygotes / the total number of retrieved oocytes × 100%.

The fertilization rate of ICSI = the number of zygotes / the number of MII stage oocytes × 100%.

The transplantable embryo rate = the number of transferable embryos (grade I–III) / the number of total embryos × 100%.

The good quality embryo rate = the number of good quality embryos (grade I–II) / the number of total embryos × 100%.

### Clinical data collection

The research team was blinded to experimental data until all clinical data had been collected from all patients. The clinical staff obtained the clinical data, including the number of oocytes retrieved, MII-stage oocytes, good quality embryos, transferable embryos, good quality embryos rate, transferable rate, and implantation outcome.

### Statistical analysis

All analyses were performed using SPSS software version 23.0 (SPSS Inc. Chicago, IL, USA). Tests of statistical significance were two sided and considered significant when *P* < 0.05. For the relationship between *ADAMTS1* or *HSPG2* mRNA level and COH outcomes, the Kolmogorov–Smirnov test was used to judge whether parameters showed continuous distribution. If so, unpaired Student’s two-tailed *t*-tests were applied. If not, non-parametric tests were applied. Analyses using non-parametric tests were marked with # in Tables 1, 2, 3. The mean ± standard error (SE) was used for the descriptive statistics of data. To determine whether *ADAMTS1* and *HSPG2* mRNA levels were associated with successful implantation, receiver operating characteristic (ROC) analyses were used to determine the predictive potential of *ADAMTS1* and *HSPG2* mRNA level on implantation. Based on the ROC curve, positive and negative predict values were calculated on the Youden index cut-off point.

**Table 1:** Patients’ characteristics of PCOS patients and normal ovulatory women

Variable	PCOS women (*N* = 45)	Normal ovulatory women (*N* = 103)	*P* value
Age (years)	28.796 ± 0.073	29.544 ± 0.034	NS
BMI (Kg/m^2^)	21.379 ± 0.056	21.776 ± 0.025	NS
Basal FSH (IU/L)	6.120 ± 0.038	6.270 ± 20.020	NS
LH (IU/L)	8.227 ± 0.096	5.015 ± 0.056	0.0104
Serum AMH (ng/mL)	10.226 ± 0.105	4.818 ± 0.022	< 0.0001
hCG dose (IU)	6130.953 ± 29.977	6868.687 ± 12.752	0.0009

Continuous variables are expressed as mean ± SE. NS, no significance; PCOS, polycystic ovarian syndrome; BMI, body mass index; FSH, follicle stimulating hormone; LH, luteinizing hormone; AMH, anti-Müllerian hormone

**Table 2:** Comparison of the baseline information and COH outcomes between low and high *ADAMTS1* expression groups in normal ovulatory women

Variable	Group with low *ADAMTS1* level (*n* = 53)	Group with high *ADAMTS1* level (*n* = 52)	*P* value
Age (years)	29.596 ± 0.072	29.490 ± 0.063	NS
BMI (Kg/m^2^)	22.104 ± 0.051	21.441 ± 0.047	NS
Basal FSH (IU/L)	6.575 ± 0.038	5.955 ± 0.042	NS
LH (IU/L)	4.089 ± 0.144	4.081 ± 0.057	NS
Serum AMH (ng/mL)	4.507 ± 0.041	5.104 ± 0.047	NS
hCG dose (IU)	6833.333 ± 24.729	6906.250 ± 26.296	NS
No. of oocytes retrieved^#^	10.981 ± 0.074	13.490 ± 0.102	0.0072
No. of matured (MII) oocytes^#^	10.692 ± 0.073	13.000 ± 0.101	0.0335
No. of transplantable embryos	5.385 ± 0.059	7.137 ± 0.077	0.0137
No. of good quality embryos^#^	2.462 ± 0.037	3.863 ± 0.053	0.0097
Transplantable rate (%)	72.872 ± 0.510	81.188 ± 0.405	NS (0.0824)
Good quality embryos rate (%)	33.360 ± 0.468	42.469 ± 0.448	NS (0.055)

Continuous variables are expressed as mean ± SE. NS, no significance; #, non-parametric tests were used

**Table 3:** Comparison of the baseline information and COH outcomes between low and high *HSPG2* expression groups in normal ovulatory women

Variable	Group with low *HSPG2* level (n = 53)	Group with high *HSPG2* level (n = 52)	*P* value
Age (years)	29.173 ± 0.064	29.922 ± 0.071	NS
BMI (Kg/m^2^)	21.608 ± 0.050	21.9401 ± 0.050	NS
Basal FSH (IU/L)	6.158 ± 0.039	6.402 ± 0.042	NS
LH (IU/L)	5.244 ± 0.135	4.745 ± 0.077	NS
Serum AMH (ng/mL)	5.129 ± 0.042	4.507 ± 0.046	NS
hCG dose (IU)	6969.388 ± 22.521	6770.000 ± 28.089	NS
No. of oocytes retrieved	12.423 ± 0.092	12.020 ± 0.092	NS
No. of matured (MII) oocytes	12.192 ± 0.091	11.471 ± 0.089	0.0474
No. of transplantable embryos	6.962 ± 0.067	5.529 ± 2.471	0.045
No. of good quality embryos	3.827 ± 0.048	2.471 ± 0.044	0.0047
Transplantable rate (%)	85.217 ± 0.311	68.600 ± 0.545	0.0004
Good quality embryos rate (%)	45.850 ± 0.444	29.734 ± 0.436	0.0005

Continuous variables are expressed as mean ± SE

## Results

### The dynamics of *ADAMTS1* and *HSPG2* mRNA levels during ovulation

**Fig. 1:**
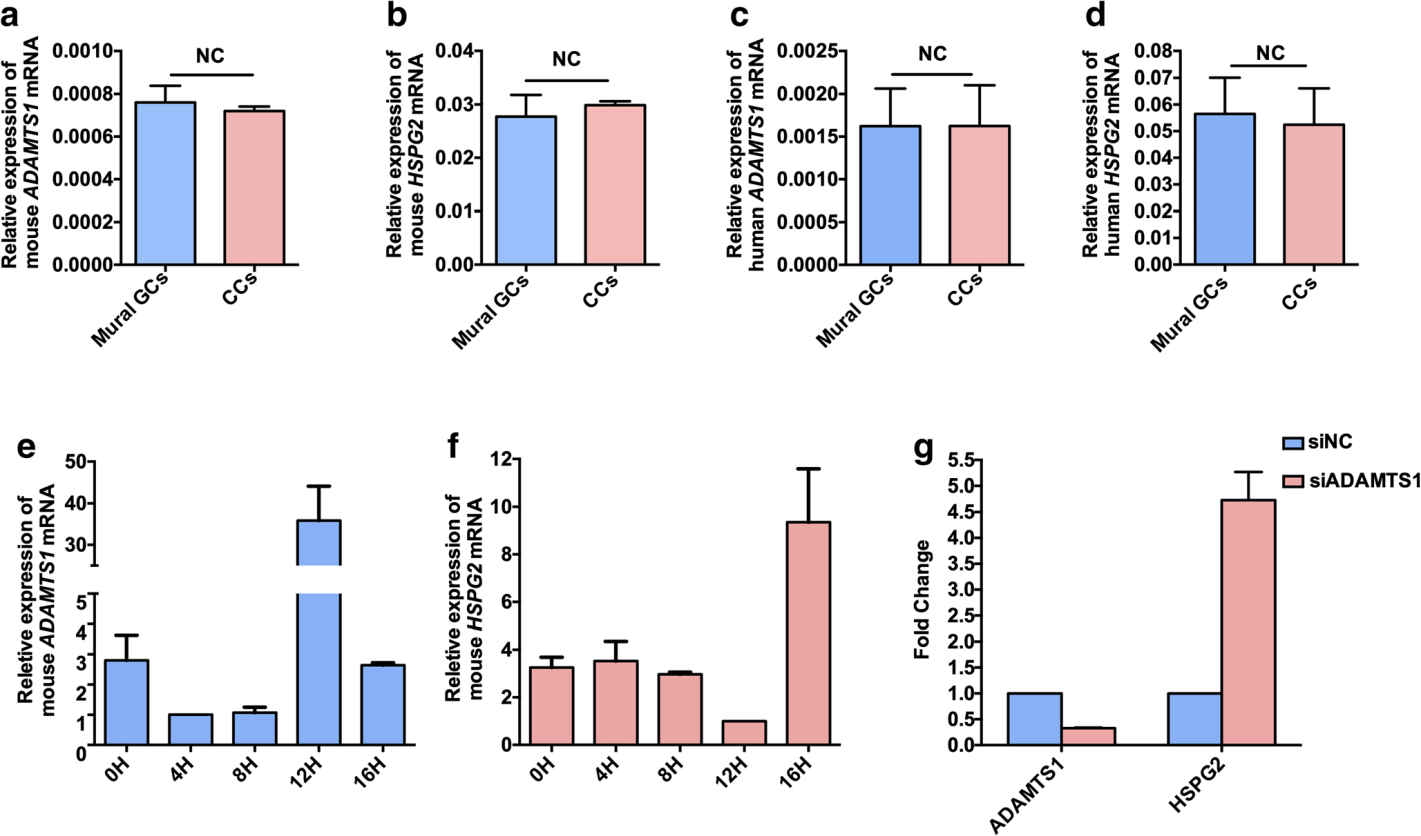
The dynamic mRNA expression of *ADAMTS1* and *HSPG2* during ovulation. (**A**–**B**) RT-PCR results show the relative mRNA expression of *ADAMTS1* and *HSPG2* in cumulus cells and mural granulosa cells from mice. (**C**–**D**) RT-PCR results show the relative mRNA expression of *ADAMTS1* and *HSPG2* in cumulus cells and mural granulosa cells from patients. (**E–F**) The relative mRNA expression of *ADAMTS1* and *HSPG2* in cumulus cells at different timepoints (0, 4, 8, 12, and 16 h) after hCG followed by PMSG treatment for 48 h. (**G**) RT-PCR analysis of the relative expression of *HSPG2* and *ADAMTS1* in cumulus cells after *ADAMTS1* RNAi treatment for 40 h. Cumulus cells were collected from 10 COCs after IVM with a negative control (NC) or *ADAMTS1* siRNAs treatment

We wished to evaluate the expression timing of *ADAMTS1* and *HSPG2* during ovulation and therefore conducted experiments in the mouse evaluating the expression of these genes following hCG injection. Human oocytes were collected at 36 h after the injection of hCG in the COH cycle, and this timepoint was equivalent to approximately 12 h in mice. The expression levels of *ADAMTS1* or *HSPG2* were comparable between mural GCs and CCs at hCG 12 h in mice or 36 h in humans (Fig. 1A–D). To evaluate the timing of the expression of *ADAMTS1* and *HSPG2* during ovulation, mouse ovarian CCs at different timepoints were collected after hCG treatment for RT-PCR. As shown in Fig. 1E, *ADAMTS1* mRNA levels were low before 8 h, sharply increased at 12 h, and then decreased to the basal level at 16 h, at which time the level at hCG 12 h was over tenfold higher compared with 0 h. In contrast to *ADAMTS1*, the mRNA level of *HSPG2* in mouse CCs decreased first and then increased, with the lowest expression at 12 h after hCG injection (Fig. 1F). This result indicates that the dynamic expression profiles of *ADAMTS1* and *HSPG2* mRNA have an inverse relationship.

### The relative expression of *HSPG2* increases after disturbing *ADAMTS1* expression in COCs

We wished to determine the effect of *ADAMTS1* expression on *HSPG2* expression and therefore conduced RNAi experiments knocking down *ADAMTS1* expression in the mouse during in vitro maturation, and CCs were collected for RT-PCR. When *ADAMTS1* mRNA was depleted in mouse CCs, the relative *HSPG2* mRNA level was significantly increased (Fig. 1G). This result indicates the essential role of *ADAMTS1* to regulate *HSPG2* mRNA levels during ovulation.

### Lower *ADAMTS1* and higher *HSPG2* mRNA levels in CCs from PCOS patients compared with normal ovulatory patients

To explore the relationship between the expression of *ADAMTS1* and *HSPG2* and COH outcomes, we compared the mRNA levels of *ADAMTS1* and *HSPG2* in the control group with tubal or male factor patients (*n* = 103) and the PCOS group (*n* = 45). Basal information of the paired groups is shown in Table 1. Patients were between 20 and 35 years old (mean ± SE: 28.796 ± 0.073 years in the PCOS group vs. 29.544 ± 0.034 years in the control group, NS). The body mass index (BMI) of subjects was between 18 and 26 kg/m^2^ in both groups (mean ± SE: 21.379 ± 0.056 kg/m^2^ in the PCOS group vs. 21.776 ± 0.025 kg/m^2^ in the control group, NS). Basal FSH was within the normal range (mean ± SE: 6.120 ± 0.038 IU/L in the PCOS group vs. 6.270 ± 0.194 IU/L in the control group, NS), whereas basal LH level in the PCOS group was nearly 1.6-fold higher than that in the control group (mean ± SE: 8.227 ± 0.096 IU/L vs. 5.015 ± 0.056 IU/L, *P* = 0.0104). Human anti-Müllerian hormone (AMH) in the PCOS group was over twofold higher compared with the control group (mean ± SE: 10.226 ± 0.105 IU/L vs. 4.818 ± 0.022 IU/L, *P* < 0.0001). Regarding the hCG dose for triggering ovulation, PCOS patients required a significantly lower dose than control patients (mean ± SE: 6130.95 ± 29.977 IU vs. 6868.69 ± 12.752 IU, *P* = 0.0009).

**Fig. 2:**
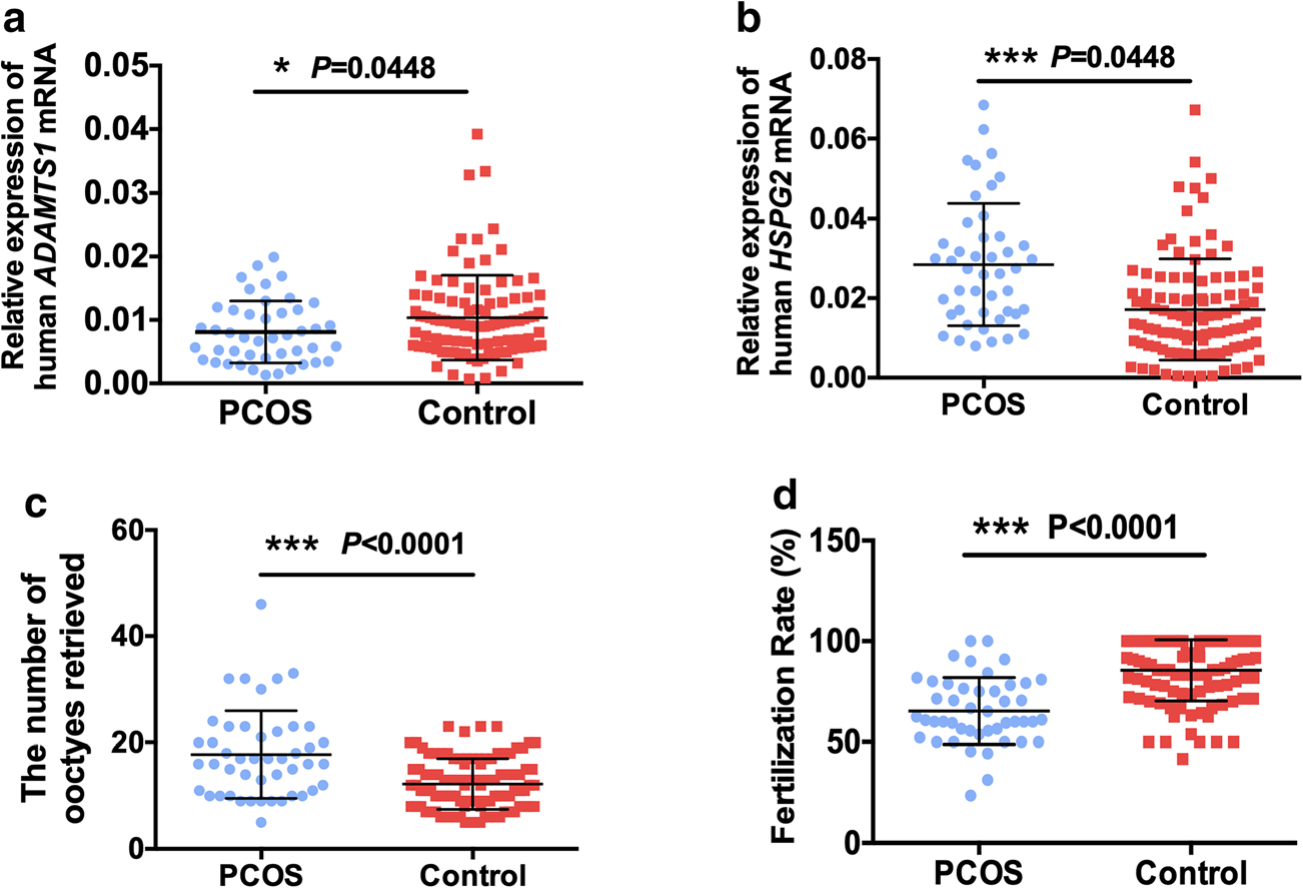
Comparison of *ADAMTS1* and *HSPG2* mRNA levels in cumulus cells from PCOS and normal ovulatory control women. (**A–B**) RT-PCR results show the relative mRNA levels of *ADAMTS1* (**A**) and *HSPG2* (**B**) in the PCOS and control groups. (**C**) The number of oocytes retrieved from PCOS patients and normal ovulatory women. (**D**) The fertilization rate was compared between the PCOS and control groups. Comparisons of the outcome with *ADAMTS1* and *HSPG2* levels in PCOS and control groups were performed using the Student’s two-tailed *t*-test or non-parametric test. Bars indicate standard deviation of the mean of all subjects in the group: *P < 0.05; ***P < 0.001

To confirm the CCs cDNA samples, we analyzed the mRNA level of *ADAMTS1*, which was reported to be expressed at a lower level in CCs derived from PCOS patients than control patients [4, 18]. In our study, the PCOS group had lower *ADAMTS1* expression in CCs than control patient CCs (0.0081 ± 0.00011 vs. 0.0104 ± 0.00006, *P* < 0.05) (Fig. 2A). In line with the inverse expression pattern of *ADAMTS1* in mice, higher *HSPG2* expression was observed in the PCOS group (0.0285 ± 0.00034 vs. 0.0172 ± 0.00012, *P* < 0.05) (Fig. 2B). Compared with the control group, we obtained more oocytes from the PCOS group (17.739 ± 0.183 vs. 12.223 ± 0.046, *P* < 0.0001) (Fig. 2C), who displayed significantly lower fertilization rates (64.245 ± 0.359% vs. 85.502 ± 0.147%, *P* < 0.0001) (Fig. 2D). These results not only further validated the inverse expression pattern in human CCs and implied that CCs derived from PCOS patients had lower *ADAMTS1* and higher *HSPG2* mRNA levels.

Because a significantly different hCG dose was used between PCOS and control patients, we verified whether hCG dose affected *ADAMTS1* and *HSPG2* expression. A subanalysis for patients with similar hCG was performed in each group. PCOS patients were divided into two groups according to the hCG dose lower or higher than the median value (hCG ≤ 5000 IU or hCG > 5000 IU). The normal ovulatory group was also divided into two groups according to the hCG dose lower or higher than the median value (hCG ≤ 7000 IU or hCG > 7000 IU). We did not find any difference in *ADAMTS1* and *HSPG2* expression, retrieved oocytes number, and fertilization rate between PCOS and control patients (Supplementary Tables 2, 3). This suggested the dose of hCG did not affect the expression of *ADAMTS1* and *HSPG2*.

### Relationship between *ADAMTS1* or *HSPG2* mRNA levels and oocyte developmental competence in normal ovulatory patients

Next, we investigated whether the *ADAMTS1* and *HSPG2* expression levels were associated with oocyte developmental competence. Thus, normal ovulatory patients were recruited and divided into two groups according to whether the *ADAMTS1* or *HSPG2* expression levels in their CCs were higher or lower than the median value of all patients (higher, *n* = 52; lower, *n* = 51). Basal characteristics (including age, BMI, basal FSH, LH, serum AMH, and hCG dose) of the paired groups divided by the *ADAMTS1* or *HSPG2* expression levels were comparable, as shown in Tables 2 and 3, which reduced the likelihood of confounding bias.

The retrieved oocyte number and matured (MII) oocyte number in patients with high *ADAMTS1* levels were significantly higher than that in patients with a low *ADAMTS1* mRNA level (13.490 ± 0.102 vs. 10.981 ± 0.074, *P* = 0.0072; 13.000 ± 0.101 vs. 10.692 ± 0.073, *P* = 0.0335) (Table 2). Regarding day 3 embryos, more transferable embryos (7.137 ± 0.077 vs. 5.385 ± 0.059, *P* = 0.0137) (Table 2) and more good quality embryos (3.863 ± 0.053 vs. 2.462 ± 0.037, *P* = 0.0097) (Table 2) were obtained from the high *ADAMTS1* group compared with the low *ADAMTS1* group (Table 2). Although a higher transferable rate and good quality embryo rate were observed in the high *ADAMTS1* group compared with the low *ADAMTS1* group, this did not reach statistical significance (81.188 ± 0.405 vs. 72.872 ± 0.510, *P* = 0.0824; 42.469 ± 0.448 vs. 33.360 ± 0.468, *P* = 0.055) (Table 2). These results suggested that *ADAMTS1* levels were linked with oocyte and embryo number and quality.

Regarding *HSPG2*, although similar oocyte numbers were retrieved from the low and high *HSPG2* groups (12.423 ± 0.092 vs. 12.020 ± 0.092, NS) (Table 3), more mature MII oocytes (12.192 ± 0.091 vs. 11.471 ± 0.089, *P* = 0.0474) (Table 3), more transferable embryos (6.962 ± 0.067 vs. 5.529 ± 2.471, *P* = 0.045) (Table 3), and more good quality embryos (3.827 ± 0.048 vs. 2.471 ± 0.044, *P* = 0.0047) (Table 3) were obtained from the low *HSPG2* group than from the high *HSPG2* group. Moreover, the transferable embryo rate and good quality embryo rate are valuable characteristics to evaluate oocyte developmental competence. The transferable rate and good quality embryo rate were significantly higher (85.217 ± 0.311% vs. 68.600 ± 0.545%, *P* = 0.004; 45.850 ± 0.444% vs. 29.734 ± 0.436%, *P* = 0.0005) in patients with lower *HSPG2* mRNA levels (Table 3). These results indicated *HSPG2* was closely associated with oocyte quality and developmental competence. Overall, the high *ADAMTS1* and low *HSPG2* expression levels were associated with better oocyte developmental competence.

### The predictive power of *ADAMTS1* and *HSPG2* mRNA levels with pregnancy outcome

ROC analysis was performed and the area under the curve (AUC) was used to estimate the accuracy of potential biomarkers. To investigate the predictive effect of the relative mRNA expression of *ADAMTS1* and *HSPG2* on COH outcome, implantation was evaluated after the IVF/ICSI procedure and embryo transfer.

**Fig. 3:**
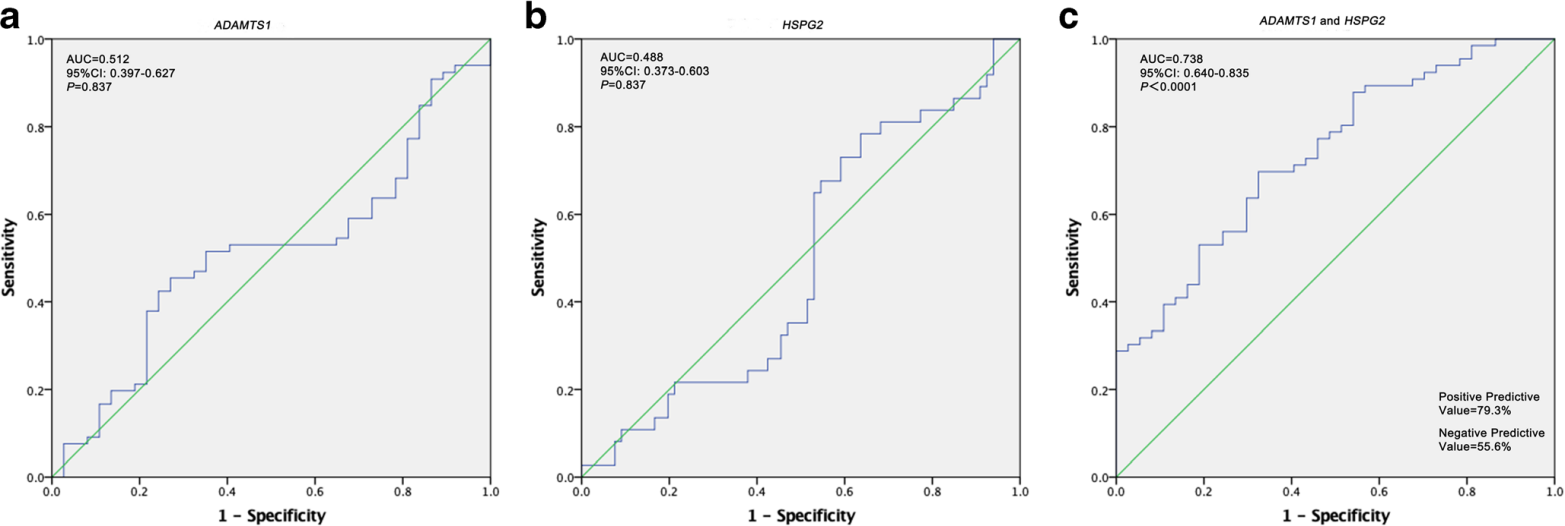
ROC curve of *ADAMTS1*/*HSPG2* mRNA levels and successful COH implantation outcomes. (**A**) ROC curve of *ADAMTS1* mRNA levels and successful COH implantation outcomes. (**B**) ROC curve of *HSPG2* mRNA levels and successful COH implantation outcomes. (**C**) ROC curve of co-measurement of *ADAMTS1*/*HSPG2* mRNA levels and successful COH implantation outcomes; AUC, area under curve; 95% CI, 95% confidence interval

Implantation was determined by serum hCG levels measured at 14 days after embryo transfer. When we analyzed the correlation of *ADAMTS1* or *HSPG2* mRNA level with implantation separately, there was no statistical significance (*ADAMTS1*: AUC = 0.512, *P* = 0.837; *HSPG2*: AUC = 0.488, *P* = 0.837) (Fig. 3A–B). Interestingly, the AUC of *ADAMTS1* and *HSPG2* to predict pregnancy was 0.738. Further calculation showed that the positive predictive value was 79.3%, and the negative predictive value was 55.6% (Fig. 3C). Therefore, we concluded that the co-measurement of *ADAMTS1* and *HSPG2* assists in the prediction of pregnancy outcomes.

## Discussion

In clinical situations, evaluating oocyte quality for IVF/ICSI and choosing which embryo to transplant are vital considerations for COH women. Previous studies have shown that *ADAMTS1*, whose expression is LH/hCG and follicle-size dependent, and which shows rapidly induced expression (>20-fold increase in CCs after hCG treatment in humans) [42, 43], was related to follicular development and ovulation and may be involved in female infertility such as PCOS [3, 7, 8, 44, 45]. PCOS patients have lower *ADAMTS1* levels in CCs and higher *ADAMTS1* levels in follicular fluids compared with normal ovulatory women [4, 25, 26]. A recent study showed that miR-375 regulated COC maturation by targeting *ADAMTS1* [46]. However, whether *ADAMTS1* is a predictor of oocyte and embryo quality in normal ovulatory patients is unclear. In addition, analysis of t a single molecule is inadequate and inaccurate to judge oocyte competence. Although previous studies have shown the co-analysis of *ADAMTS1* and versican improved accuracy [14, 20, 22], our study investigated the use of a new molecule to predict high quality oocytes.

Proteoglycans consist of a protein core and covalently attached glycosaminoglycan (GAG) chains, which are basic components of the ECM [13]. Proteoglycans such as versican, aggrecan, perlecan, laminin, prolargin, and collagen type IV exist in ovarian follicles [26]. Versican and aggrecan, but not perlecan, are confirmed substrates of *ADAMTS1* [25]. There is limited evidence for the downregulation of *HSPG2* expression at the mRNA level by *ADAMTS1*. However, studies reported that perlecan in follicular fluids reached a peak at 12 h after injecting hCG [47] and may have a close relationship with oocyte developmental competence [48–50]. Because perlecan in follicular fluids is derived from GCs, it will be interesting to investigate correlations between *HSPG2* mRNA levels in human CCs and oocyte developmental competence. This study focused on both PCOS patients and normal ovulatory COH women, and we found that patients with lower *HSPG2* mRNA levels in CCs had better COH outcomes.

To date, most studies have focused on the pre-embryo transfer period, including the mature oocyte and fertilization rate, but no study has examined the predictive power of *ADAMTS1/HSPG2* mRNA levels in human CCs with oocyte quality, especially during clinical pregnancy. This study extended the follow-up time to implantation.

We compared the relative expression of *ADAMTS1* in human CCs between PCOS and normal ovulatory women and found lower *ADAMTS1* expression in PCOS as previously reported [4]. These findings also confirm a study by Brown et al. that reported ovulation and subsequent fertilization were seriously impaired in *ADAMTS1*-deficient mice [14]. At the same time, we found that *HSPG2* mRNA levels were significantly higher in CCs from PCOS patients than in control patients. After COH, the PCOS group had low *ADAMTS1* and high *HSPG2* levels with a significantly low fertilization rate, although more oocytes were retrieved for pregnancy from PCOS patients.

During ovulation in mice, we found that the expression of *ADAMTS1* or *HSPG2* mRNA were comparable between mural and cumulus cells derived from mice or humans around the time of ovulation. Therefore, in this study, easily harvested CCs were used to evaluate the expression of *ADAMTS1* and *HSPG2*. Of note, the levels of *ADAMTS1* and *HSPG2* were inversely correlated, especially at hCG 12 h in C57BL/6 mice, a timepoint near ovulation. Moreover, *HSPG2* was significantly increased after downregulating *ADAMTS1*. Therefore, we speculate that *ADAMTS1* and *HSPG2* may have related functions during ovulation.

To date, no studies have reported a relationship between *ADAMTS1* and *HSPG2* expression and COH outcomes in normal ovulatory women. Therefore, we detected the mRNA levels of *ADAMTS1* and *HSPG2* in tubal or male factor patients. Patients were divided into two groups based on *ADAMTS1* or *HSPG2* expression levels higher or lower than the median value (low and high), to analyze the relationship between *ADAMTS1* or *HSPG2* expression and COH outcomes. In tubal or male factor patients, the group with higher *ADAMTS1* levels and lower *HSPG2* levels had a better clinical outcome, with more MII stage oocytes, transferable embryos, good quality embryos, and a higher rate of transferable embryos or good quality embryos. Our results indicate that the relative expression of *ADAMTS1* was correlated positively with the number of oocytes and embryos, while the relative expression of *HSPG2* correlated positively with the quality of oocytes and embryos. Therefore, the co-measurement of *ADAMTS1* and *HSPG2* might better predict COH outcomes and later analysis demonstrated this.

ROC analysis was performed, and the AUC was used to estimate the accuracy of potential biomarkers, including *ADAMTS1* mRNA with implantation, *HSPG2* mRNA with implantation, and co-measurement of *ADAMTS1* mRNA and *HSPG2* mRNA with implantation. Interestingly, the co-measurement of two genes improved the predictive power.

In this study, *HSPG2* seemed more significant than *ADAMTS1* at predicting oocyte and embryo quality. Because perlecan has a critical role in numerous physiological processes [22], the relative expression of *HSPG2* mRNA may be more useful than that of *ADAMTS1 to* predict good quality embryos. For example, Princivalle et al. reported that perlecan provided attachment for anticoagulant heparan sulfate proteoglycans (aHSPGs) on the cell surface and in the extracellular matrix, which contributed to the control of proteolytic activity involved in follicular development and ovulation [51]. Abnormal *HSPG2* encodes too much or too little perlecan, which may influence the interaction with aHSPGs and *ADAMTS1*, while at the same time, it may affect endothelial vascular growth during follicular development [3, 8, 10, 24, 44].

Substantial evidence has shown that EGF-like factors (*AREG, EREG, BTC*), EGFR and the ERK1/ERK2 signaling cascade have critical roles for cumulus expansion, oocyte maturation, and follicle rupture. Many genes related to ovulation including *AREG, EREG, HAS2, PTGS2, CITED2, CITED4*, and *ADAMTS1* were induced by LH via the ERK1/ERK2 signaling cascade [6, 52–54]. These gene expressions may be essential for oocyte meiotic maturation. For example, the spindles of MII oocytes from *AREG* knockout mice were abnormal [55]. Previous studies have confirmed that the expression of some of these genes in GCs including AREG, EREG, and CITED2 might be potential biomarkers to predict human oocyte quality in IVF/ICSI [1, 23, 56–58].

And *HSPG2*/perlecan is important for chondrocyte clustering and mediates its effect through the ERK pathway [59]. Based on these studies and our research, the ERK pathway may be involved in the mechanism of how *ADAMTS1/HSPG2* affect oocyte quality. Co-measurement of *ADAMTS1/HSPG2* mRNAs in human CCs might be a predictive method for human oocyte quality. We are interested in exploring further the correlation between the mRNA levels of these genes in single denuded MII oocytes and clinical outcomes.

In conclusion, we shed light on the expression of *ADAMTS1* and *HSPG2* in mural GCs and CCs in mice and humans and found that high *ADAMTS1* levels and low *HSPG2* levels in CCs were associated with high oocyte quality and COH outcomes in both PCOS and normal ovulatory women. Co-measurement of *ADAMTS1* and *HSPG2* improved the predictive power to estimate clinical pregnancy outcomes. Detection of *ADAMTS1* and *HSPG2* mRNAs in human CCs may help doctors make decisions in specific cases, such as patients who have failed multiple transplants. However, prior to use for clinical diagnosis, evidence to validate the close correlation between *ADAMTS1* and *HSPG2* mRNA levels and pregnancy outcomes (such as implantation rate and live birth rate) in other or larger populations is required. This study only identified the correlation between *ADAMTS1* and *HSPG2* expression levels and COH outcomes. Whether low *ADAMTS1* expression is the cause of anovulation in PCOS patients needs to be investigated further. Additional studies are also needed to clarify how *ADAMTS1* influences the expression of *HSPG2.*

## Electronic supplementary material

ESM 1 (DOCX 17.8 kb)
