# Supplementary material for: ADAMTS1 and HSPG2 mRNA levels in cumulus cells are related to human oocyte quality and controlled ovarian hyperstimulation outcomes
Source: J Assist Reprod Genet. 2020 Jan 23;37(3):657–67. doi: 10.1007/s10815-019-01659-8 (PMC7125252; doi:10.1007/s10815-019-01659-8)
Supplement: Supplementary file 1 — (DOCX 17.8 kb) [file 10815_2019_1659_MOESM1_ESM.docx]

Supplementary Table I. Real-Time PCR primer Sequence

| Gene | Species | Primers Sequence |
| --- | --- | --- |
| *Actb* | Mouse | Forward, 5’-CCGTAAAGACCTCTATGCC-3’ |
|  |  | Reverse, 5’- CTCAGTAACAGTCCGCCTA-3’ |
| *Adamts1* | Mouse | Forward, 5’- AAGGAAGAAGCGATTTGTGTCC-3’ |
|  |  | Reverse, 5’- CCACCGAGAACAGGGTTAGA-3’ |
| *Hspg2* | Mouse | Forward, 5’-TGGAGCCCGAATACAGGAAGA-3’ |
|  |  | Reverse, 5’-AGATCCGTCCGCATTCCCT-3’ |
| *ACTB* | Human | Forward, 5’-GCAAAGACCTGTACGCCAA-3’ |
|  |  | Reverse, 5’-GGAGGAGCAATGATCTTGATCTTC-3’ |
| *ADAMTS1* | Human | Forward, 5’-AAGACGAGGACGAAGGGACT-3’ |
|  |  | Reverse, 5’-TAGCGGTGACTGGACACAAA-3’ |
| *HSPG2* | Human | Forward, 5’-CCAGCTCTCTTTTGGCAACT-3’ |
|  |  | Reverse, 5’-GGTGTATCGCAACTTCCCAC-3’ |

Supplementary Table II. Comparison of the *ADAMTS1* and *HSPG2* mRNA level in groups lower or higher than the median of hCG doses (hCG≤5000IU or hCG>5000IU) in PCOS patients.

| Variable | hCG≤5000IU  (N=23) | hCG>5000IU  (N=22) | *P* value |
| --- | --- | --- | --- |
| Relative Expression of *ADAMTS1* | 0.0078 ± 0.00023 | 0.0084 ± 0.00021 | NS |
| Relative Expression of *HSPG2* | 0.0282 ± 0.00077 | 0.0287 ± 0.00059 | NS |
| The number of oocytes retrieved | 18.522 ± 0.311 | 16.955 ± 0.415 | NS |
| Fertilization Rate, % | 62.968 ± 0.725 | 67.391 ± 0.713 | NS |

Continuous variables are expressed as mean ± SE. NS, no significance.

Supplementary III. The comparison of ADAMTS1 and HSPG2 mRNA level in groups lower or higher than the median of hCG doses (hCG≤7000IU or hCG>7000IU) in normal ovulatory women.

| Variable | hCG≤7000IU  (N=52) | hCG>7000IU  (N=51) | *P* value |
| --- | --- | --- | --- |
| Relative Expression of *ADAMTS1* | 0.0169 ± 0.00025 | 0.0174 ± 0.00025 | NS |
| Relative Expression of *HSPG2* | 0.0107 ± 0.00016 | 0.0100 ± 0.00008 | NS |
| Number of oocytes retrieved | 12.635 ± 0.095 | 11.804 ± 0.088 | NS |
| Fertilization Rate (%) | 85.690 ± 0.281 | 85.310 ± 0.300 | NS |

Continuous variables are expressed as mean ± SE. NS, no significance.
